# Frequency, Origins, and Evolutionary Role of Chromosomal Inversions in Plants

**DOI:** 10.3389/fpls.2020.00296

**Published:** 2020-03-18

**Authors:** Kaichi Huang, Loren H. Rieseberg

**Affiliations:** Department of Botany and Biodiversity Research Centre, University of British Columbia, Vancouver, BC, Canada

**Keywords:** inversions, comparative genomics, reduced recombination model, secondary contact, comparative genetic mapping

## Abstract

Chromosomal inversions have the potential to play an important role in evolution by reducing recombination between favorable combinations of alleles. Until recently, however, most evidence for their likely importance derived from dipteran flies, whose giant larval salivary chromosomes aided early cytogenetic studies. The widespread application of new genomic technologies has revealed that inversions are ubiquitous across much of the plant and animal kingdoms. Here we review the rapidly accumulating literature on inversions in the plant kingdom and discuss what we have learned about their establishment and likely evolutionary role. We show that inversions are prevalent across a wide range of plant groups. We find that inversions are often associated with locally favored traits, as well as with traits that contribute to assortative mating, suggesting that they may be key to adaptation and speciation in the face of gene flow. We also discuss the role of inversions in sex chromosome formation, and explore possible parallels with inversion establishment on autosomes. The identification of inversion origins, as well as the causal variants within them, will advance our understanding of chromosomal evolution in plants.

## Introduction

Species and ecotypes are often differentiated by chromosomal rearrangements, such as translocations and inversions. The latter were first discovered by Sturtevant (1921) when comparing genetic linkage maps of closely related *Drosophila* species. Sturtevant further deduced that inversions reduce the rate of recombination in heterozygotes (which is key to their main evolutionary role), and validated this claim through observations of the giant larval salivary chromosomes found in *Drosophila*. Inverted regions were subsequently identified from banding patterns of chromosomes in many other species and became the first genetic markers used to reconstruct phylogenies (Krimbas and Powell, 1992). The abundance of inversion polymorphisms detected in these studies also inspired population geneticists to investigate patterns of variation within and between species of *Drosophila* (Dobzhansky, 1970).

Until recently, most evidence regarding the frequency and evolutionary role of inversions came from studies of Dipteran systems, such as *Drosophila* (Noor et al., 2001; Ortiz-Barrientos et al., 2002), *Anopheles* (Ayala and Coluzzi, 2005; Ayala et al., 2010) and *Rhagoletis* (Feder et al., 2003a, b). This was partly due to the ease of identifying inversions in Dipteran salivary gland chromosomes, but also because of the widespread recognition of the importance of inversions in this group. Over the past two decades, however, comparative genetic mapping and genomic approaches have revealed that inversions are ubiquitous across the plant and animal kingdoms, either fixed between or polymorphic within species (Wellenreuther and Bernatchez, 2018). Detailed studies of the genetic contents and establishment of inversions have not only confirmed a longstanding hypothesis that inversions play an important role in adaptation by reducing recombination between favorable combinations of alleles (Kirkpatrick and Barton, 2006; Lowry and Willis, 2010), but also that they contribute to speciation in a similar way - by suppressing recombination between local adapted alleles and those causing assortative mating (Trickett and Butlin, 1994).

In this paper, we first review various approaches that have been employed to detect inversions within and between plant species, as well as studies that report on inversion abundance across a wide range of plant groups. We then discuss possible scenarios for the origin and spread of inversions inspired by theoretical and empirical studies. We further illustrate their important role in speciation with case studies that have associated inversions with traits known to underlie ecological adaptation and reproductive isolation. We also discuss the role of inversions in sex chromosome formation and whether the stepwise establishment of inversions seen on sex chromosomes might also occur on autosomes. Finally, we suggest avenues for future studies to bridge gaps in our understanding of the evolutionary role of inversions in plants.

## Detection of Inversions and Their Pervasiveness in Plants

### Cytogenetic Studies

Most early evidence of inversions in plants came from two sources. First, as alluded to above, inversions could sometimes be inferred from the chromosome banding patterns seen in karyotypes (Greilhuber and Speta, 1976; Konishi and Linde-Laursen, 1988; Rodriguez et al., 2000). This approach worked well in plants with small numbers of large and distinctive chromosomes, but was impractical in most species of plants. Also, even when feasible, only very large inversions were typically detectable. Nonetheless, these karyotypic analyses indicated that inversions were not uncommon in plants.

**FIGURE 1:**
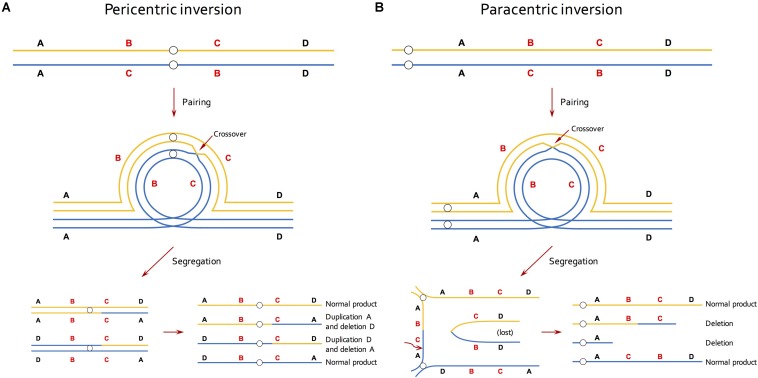
Effective reduction in recombination of inversion by selection against recombinant gametes in meiosis. **(A)** In individuals that are heterozygous for a pericentric inversion, a single crossover within the inversion generates unbalanced gametes that contain a duplication and a deletion. **(B)** In individuals that are heterozygous for a paracentric inversion, a single crossover within the inversion produces a dicentric bridge and an acentric fragment. The acentric fragment is lost because it cannot be drawn to either end and the chromosomal bridge breaks at random point during segregation, resulting in two deletion products. Lines of blue and orange colors represent homologous chromosomes and small circles indicate centromeres.

A second source of information about inversions came from studies of chromosome pairing in meiosis (Lewis and Roberts, 1956; Sybenga, 1975; Ahmad et al., 1979; Gopinathan and Babu, 1986; Anderson et al., 2010). This approach relied on that fact that crossing over in inversion heterozygotes creates distinctive meiotic configurations (Sybenga, 1975; Figure 1). While such an approach is feasible in taxa with small and/or morphologically similar chromosomes, it under-estimates inversion abundance because recombination within inversions is required for their detection. Thus, small inversions, or those in low-recombining regions of the genome, will be missed.

In recent decades, *in situ* hybridization approaches have been widely employed to study karyotype evolution within and between species. Such approaches are most powerful in groups such as the Brassicaceae and Solanaceae, in which virtually repeat-free BAC contigs covering much of the genome are available for use as probes, permitting “comparative chromosome mapping” (Lysak and Lexer, 2006). Successful application of this method has led to the discovery of numerous inversions across various clades of the Brassicaceae, but especially *Arabidopsis* and *Brassica* (Lysak et al., 2006, 2007; Mandakova and Lysak, 2008; Mandakova et al., 2015; Lee et al., 2017), as well as among and within species in *Solanum* (Szinay et al., 2012). However, suitable sets of chromosome-specific painting probes are needed for the broader application of this approach in other plant groups.

### Comparative Genetic Mapping

With the development of DNA markers in the latter part of the 20th century, it became feasible to develop genetic linkage maps that were sufficiently dense to permit detection of chromosomal rearrangements between plant genomes. More recently, advances in high throughput sequencing and computational methods permit high-resolution genetic mapping and inference of large structural variants from low coverage sequence data (e.g., Flagel et al., 2019). Comparative genetic mapping has been broadly applied, especially in crop rich families such as Solanaceae, Poaceae, and Brassicaceae. While successful, the number of rearrangements detected depends in part on marker density and recombination rates. If either is low, then rearrangements will be missed. We summarize a few well known examples below, both to illustrate that inversions are common in essentially all plant taxa analyzed, but also for comparison to genomic studies (below), which show that comparative mapping, like chromosome banding and meiotic analyses, greatly under-estimated inversion numbers.

In Solanaceae, for example, the genomes of potato and tomato were found to differ by only five paracentric inversions (Bonierbale et al., 1988; Tanksley et al., 1992), while at least 19 inversions and 6 chromosome translocations differentiate potato and pepper (Wu et al., 2009; summarized in Szinay et al., 2012). In Poaceae, genetic maps based on restriction fragment length polymorphism markers identified an inversion on the short arm of chromosome 9 between sorghum and maize, which also differentiates maize from its close relative *Zea mexicana* (Berhan et al., 1993). Likewise, Ahn and Tanksley (1993) showed that while the rice and maize genomes are largely conserved in gene order, multiple inversions and translocations occurred after the polyploidization of maize. Analyses of different intraspecific maps of *Brassica oleracea* revealed that small inversions among morphotypes were the most frequent form of rearrangements followed by translocations (Kianian and Quiros, 1992). A comparison between *Arabidopsis thaliana* and *Brassica nigra* also identified numerous translocations and even more inversions between the two genera (Lagercrantz, 1998).

The comparative mapping approach has also been applied to ecological and evolutionary model systems, including *Mimulus*, *Populus*, and *Helianthus*. This work has identified numerous chromosomal rearrangements from the ecotypic to interspecific level. For example, Lowry and Willis (2010) discovered an inversion between annual and perennial ecotypes of *Mimulus guttatus* by comparing maps from multiple F2 mapping populations. Using a similar approach, Fishman et al. (2013) identified two reciprocal translocations and three inversions between *Mimulus cardinalis* and *M. lewisii*. Using evidence from both linkage and physical maps, possible inversions were also inferred between *Populus* species (Drost et al., 2009; Tong et al., 2016). In *Helianthus*, low density linkage maps of *Helianthus annuus* and *H. petiolaris* suggested that three inversions and as many as eight translocations differentiated the species (Rieseberg et al., 1995; Burke et al., 2004). However, a recent follow-up study (Ostevik et al., 2019), which employed higher density genetic maps and a novel algorithm for synteny block detection, found 50-60 inversions between the species and 6-8 translocations. Thus, low-density maps appear better able to detect translocations than inversions, presumably because detection of the former requires fewer markers and is less sensitive to marker ordering errors. Ostevik et al. (2019) also applied their algorithm to comparisons of new maps for the two subspecies of *H. petiolaris*, as well as high-density genetic maps previously published for three other species (Barb et al., 2014). Up to 74 inversions and 15 translocations were found across the five taxa. Lastly, Huang et al. (2019) developed genetic maps for dune and non-dune ecotypes of *H. petiolaris* and successfully identified multiple inversions, but no translocations.

### Comparative Genomic Studies

In recent years, the ever-increasing number of high quality genome assemblies and other genomic datasets have greatly facilitated the detection of chromosomal rearrangements and uncovered very large numbers of inversions between closely related species (Table 1). For example, a *de novo* assembly of *Arabidopsis thaliana* L*er*-0 strain revealed 47 inversions between its genome and that of the widely used Col-0 accession (Zapata et al., 2016), although some unknown fraction of these inversions might have been introduced by mutagenesis. A reference genome of *Arabidopsis lyrata* was compared to that of *A. thaliana*, and 154 inversions were identified, as well as two reciprocal translocations and three chromosomal fusions previously revealed by genetic mapping (Yogeeswaran et al., 2005; Hu et al., 2011). Several inversions were also found between cucumber and melon (Huang et al., 2009; Garcia-Mas et al., 2012), and five paracentric and one pericentric inversions were revealed between cultivated and wild cucumber with the aid of comparative fluorescence *in situ* hybridization (Yang et al., 2012). Whole-genome sequencing of pepper confirmed previously reported large inter-chromosomal translocations and identified 367 inversions between pepper and potato (Qin et al., 2014), about 20x more than were identified via comparative mapping. In addition, a total of 214 inversions were identified between rice (*Oryza sativa*) and its close relative *O. brachyantha* (Chen et al., 2013). And a comprehensive study using homologous gene sequences showed that short paracentric inversions and short intra-chromosomal translocations were the most common rearrangements in the grass family Poaceae (Dvorak et al., 2018). Other well characterized examples come from comparisons of different cultivars of cotton (Yang et al., 2019) and grapevine (Zhou et al., 2019); details in Table 1.

**TABLE 1:** Summary of inversions from comparative genomics studies.

Species	Common name	Number of inversions	Size	Number of translocations or fusions	Divergence time (MYA)	Evolutionary rate (inversion/MYA)	References
*Arabidopsis thaliana* strains	Thale cress	47	115 bp-1.17 Mbp	0	−	−	Zapata et al., 2016
*Arabidopsis lyrata/A. thaliana*	Thale cress/lyrate rockcress	154	−	5	10	15.4	Hu et al., 2011
*Cucumis sativus*	Wild and cultivated cucumbers	5 paracentric, 1 pericentric	−	0	−	−	Yang et al., 2012
*C. sativus/Cucumis melo*	Cucumber/melon	Several	−	−	10	−	Huang et al., 2009; Garcia-Mas et al., 2012
*Capsicum annuum/Solanum lycopersicum*	Pepper/tomato	468	−	612	20	23.4	Qin et al., 2014
*Capsicum annuum/ Solanum tuberosum*	Pepper/potato	367	−	430	20	18.35	
*Oryza sativa/O. brachyantha*	Rice	214	−	0	15	14.27	Chen et al., 2013
*Gossypium hirsutum*	Upland cotton	60	−	1314	−	−	Yang et al., 2019
*Gossypium hirsutum*/*G. arboreum*	Cottons	39	−	35	1-2	18.5-39	
*Gossypium hirsutum*/*G. raimondii*	Cottons	15	−	29	1-2	7.5-15	
*Vitis vinifera* cultivars	Grapevine	1513	−	3786	−	−	Zhou et al., 2019
*Aegilops tauschii*	Tausch’s goatgrass	44	1.6–8.0 Mbp	−	3	14.67	Dvorak et al., 2018
*Triticum turgidum* subgenome A	Wild emmer wheat	91			3	30.33	
*Triticum turgidum* subgenome B		65			3	21.67	
*Brachypodium distachyon*	Purple false brome	82			35	2.34	
*Oryza sativa*	Rice	20			47	0.43	
*Sorghum bicolor*	Sorghum	33			53	0.62	

Using the number of inversions reported in these studies and species’ divergence times obtained from the literature, we estimated the rate of inversion evolution to be about 15–30 inversions per million years (Table 1). However, this estimate should be treated with caution since the quality of genome assemblies and the methods employed to identify chromosome rearrangements varied among studies. Also inversion sizes typically were not reported in these studies, except for Zapata et al. (2016) and Dvorak et al. (2018), who showed that most inversions are small in size. Some of the variation in rates of inversion evolution reported for different groups likely derives from different size cut-offs used to report inversions. For example, the range of inversion sizes reported by Zapata et al. (2016) and Dvorak et al. (2018) are almost completely non-overlapping (Table 1). In the future, it would be useful if studies would report the size distributions of inversions, as well as the extent of sequence divergence between inversion haplotypes.

### Population Genomic Approaches

While these comparative approaches offer a means for determining the number of inversions between species, they typically tell us little about the distribution of inversion polymorphisms within species or the traits that are associated with the inversions. However, this information is needed to understand how inversions are established, as well as their role in adaptive evolution and speciation. Fortunately, two population genomic approaches have recently been developed that permit inference of inversions from resequencing data when paired with high quality reference sequences.

One approach detects potential inversions by scanning the genome for regions of high linkage disequilibrium (LD) among linked markers (Kemppainen et al., 2015). The rationale for this approach is that recombination suppression should produce very high LD among SNPs within inversions. Although gene conversion and double recombination can break down LD in the middle of old and large inversions, such as that observed for some inversions in *Drosophila* (Korunes and Noor, 2019), whether this is common in plants is unclear. Other mechanisms that reduce recombination, such as pericentromeric heterochromatin, will also lead to high LD regions. Thus, the LD scan should be complemented by an analysis of genotypic relationships within the predicted inversion using principle component analysis (PCA) or a similar method. Because inversions only suppress recombination in heterozygotes, three distinct genotypic clusters should be detected within an inversion representing each inversion orientation (0/0, 1/1), plus heterozygotes between inversion haplotypes (0/1). While LD scans have been employed to search for inversions in animals (Faria et al., 2019), we are unaware of their application to plants.

A second approach takes advantage of the effects of inversions on population structure. This approach assumes that the lack of gene flow between inversion haplotypes will result in differences in patterns of genetic relatedness between inverted and collinear regions. These outlier regions can be detected by conducting windowed analyses of population structure across the genome, such as that implemented by the Local PCA/population structure (lostruct) program developed by Li and Ralph (2019). As with the LD method, analyses of genotypic relationships within the predicted inversion can offer further support for the putative inversion. The local population structure approach has been used to detect polymorphic inversions from RAD sequencing data or whole-genome shotgun data within the wild sunflower species, *Helianthus annuus*, *H. argophyllus*, and *H. petiolaris* (Huang et al., 2019; Todesco et al., 2019). While many of the inversions predicted by this method in sunflower have been subsequently confirmed via comparisons of reference sequences, comparative mapping, or Hi-C sequencing, two were not, indicating that these population genomic approaches can offer suggestive evidence of inversions, but are less diagnostic. Other mechanisms, such as recent introgression, could generate patterns similar to those of an inversion. Conversely, small inversions, or inversions lacking elevated population structure or high LD outside of inversion breakpoints, might not be detected by these methods.

Using these population genomic approaches, the genotypes of multiple individuals can be simultaneously determined for all detected inversions, which provide useful information on their frequency and geographic distribution. Mapping the breakpoints of inversions will also be helpful for developing PCR markers to determine patterns of inversion polymorphism across large numbers of individuals. This sets the stage for associating traits and environmental factors with inversion haplotypes, thereby revealing the evolutionary forces that shape the pattern of inversion polymorphisms. Although additional independent lines of evidence are encouraged to confirm putative inversions suggested by such methods, population genomic approaches, coupled with ever-expanding population sequencing data, have great potential to further our understanding of the prevalence and evolutionary role of inversions in plants, especially in non-model species.

### Different Likelihood of the Establishment of Inversions and Translocations

As discussed above, comparative mapping of plant species typically identified more inversions than inter-chromosomal translocations or “fusions” (terminal reciprocal translocations). In general, as mapping or sequencing resolution increases, so does the number of inversions detected (Wu and Tanksley, 2010; Hu et al., 2011; Chen et al., 2013; Qin et al., 2014; Ostevik et al., 2019). In contrast, little or no increase in the numbers of inter-chromosomal translocations is reported with increasing resolution. Some studies (not discussed here) have focused exclusively on chromosomal-scale translocations and fusions/fissions or did not clearly differentiate rearrangement types and thus are not relevant to this question.

We suspect that variation in the abundance of major inter-chromosomal translocations versus inversions relates more to differences in the likelihood of their establishment than to variation in mutation rates. Translocation heterozygotes involving different chromosomes will show mis-segregation during meiosis and produce unbalanced and inviable gametes (King, 1993). This strong heterozygous disadvantage (underdominance) of inter-chromosomal translocations makes them difficult to establish. On the other hand, plants seem to be more tolerant of intra-chromosomal rearrangements such as inversions. While recombination between inversion orientations is predicted to result in inviable gametes, the evidence for this is surprisingly sparse and comes mainly from interspecific crosses. Meiotic abnormalities diagnostic of inversions, along with reduced pollen viability, have been reported, for example, in hybrids of *Gibasis venustula* and *G. speciose* (Kenton, 1981), *Vigna umbellate* and *V. minima* (Gopinathan and Babu, 1986), as well as between races of *Paspalum notatum* (Stein et al., 2004), but the fertility loss is typically much smaller than for most translocations. Surprisingly, inversions segregating within species often have no visible effect on fertility, such as reported for *Brassica oleracea* (Kianian and Quiros, 1992) and maize (Fang et al., 2012). In *Mimulus* and *Helianthus*, crosses between ecotypes that are separated only by inversions do not show reduced pollen viability (Lowry and Willis, 2010; Ostevik et al., 2016; Huang et al., 2019), although meiotic abnormalities diagnostic for inversions have been reported for interspecific crosses (Chandler et al., 1986). This suggests that the reduction in recombination associated with inversions within plant species is typically achieved by disrupting pairing and crossing over between inverted regions (Searle, 1993) rather than selection against inviable recombinant gametes. Regardless of the cause, the minimal underdominance of many inversions should ease their establishment.

In a number of comparative genomic studies, more translocations were reported than inversions (Table 1). Variation in the abundance of inversions and translocations seen in Table 1 stems partly from differences in methods, criteria (e.g., size cut-offs), and power for detecting structural variants, as opposed to real differences in their frequency. Some studies (Yang et al., 2019; Zhou et al., 2019) applied whole-genome alignment, long-read alignment and short-read alignment to detect both inter- and intra-chromosomal translocations of various sizes (transposed genomic segments), while others (Hu et al., 2011) have focused exclusively on large inter-chromosomal reciprocal translocations. More robust conclusions about the prevalence of inversions and translocations will not only require that studies be more parallel in terms of data and methodology employed, but also that they take rearrangement size into account.

## Origin and Establishment of Inversions

There are a number of different molecular mechanisms by which inversions can arise, including ectopic recombination between copies of repeated sequences such as transposable elements, tRNA genes or segmental duplications, or by chromosomal breakage and repair by non-homologous end-joining (Gray, 2000; Feschotte and Pritham, 2007; Delprat et al., 2009). Both mechanisms have been shown to occur in plants, especially in maize (Lister et al., 1993; Ziolkowski et al., 2003; Zhang and Peterson, 2004; Yu et al., 2011; Knoll et al., 2014). Epigenetic modification, given its role in transposable element de-activation and heterochromatin formation, may also play an important role in chromosome evolution in plants (Li et al., 2017). Given the high fraction of plant genomes occupied by transposable elements and other duplicated sequences, inversion mutation rates are likely to be high. However, the relative importance of these different mechanisms and the overall incidence of inversions in natural populations remain to be explored.

Like other genetic mutations, inversions can change in frequency as a consequence of genetic drift or selection. Early models of chromosomal evolution assumed that most underdominant rearrangements became established through drift (White, 1973; Lande, 1979). However, the fixation of a strongly underdominant mutation through drift is unlikely, except under extreme conditions, such as can be found in small founder populations and/or through inbreeding. The conditions for fixation are relaxed for neutral or weakly underdominant mutations such as inversions. Nonetheless, the fact that inversions are frequent in outcrossing species with large effective population sizes, co-vary with ecological variation, and underlie important adaptive traits (Hoffmann and Rieseberg, 2008; Lowry and Willis, 2010; Todesco et al., 2019), suggests that the establishment and spread of large inversions is most likely driven by natural selection. The jury is still out for the many small inversions that differentiate plant genomes.

Meiotic drive has been proposed as another possible mechanism for the fixation of chromosomal rearrangements. While meiotic drive may very well explain the establishment of large inter-chromosomal translocations, it appears to be too infrequent to account for the abundance of inversions seen both within and between species (Coyne, 1989). It also has been hypothesized that inversions could be favored if breakpoints disrupt an open reading frame or alter gene expression (Hoffmann and Rieseberg, 2008). While we are unaware of a case in plants where such changes have been shown to be adaptive, it is important to keep in mind that few breakpoints have been characterized for inversions with clear phenotypic effects. In the best-studied examples in plants (or animals), selection favoring the establishment of inversions appears to arise indirectly from their impact on reducing recombination within the inverted region. Thus, most recent evolutionary models for the establishment of inversions have focused on this property (Kirkpatrick and Barton, 2006; Burger and Akerman, 2011; Feder et al., 2011; Charlesworth and Barton, 2018).

The importance of recombination to the establishment and spread of inversions was initially put forward by Dobzhansky (1970) based on studies of *Drosophila*, in which inversions typically have little impact on fertility. Dobzhansky argued that genes within inversions were co-adapted, meaning that the fitness of the alleles held together by the inversion would be greater than the sum of their independent effects. A newly arisen inversion carrying a co-adapted set of alleles would spread to fixation in a population unless it was under balancing selection or there was ongoing migration from other populations. Unfortunately, we do not know whether such co-adaptation (i.e., favorable epistatic interactions) is common within inversions.

**FIGURE 2:**
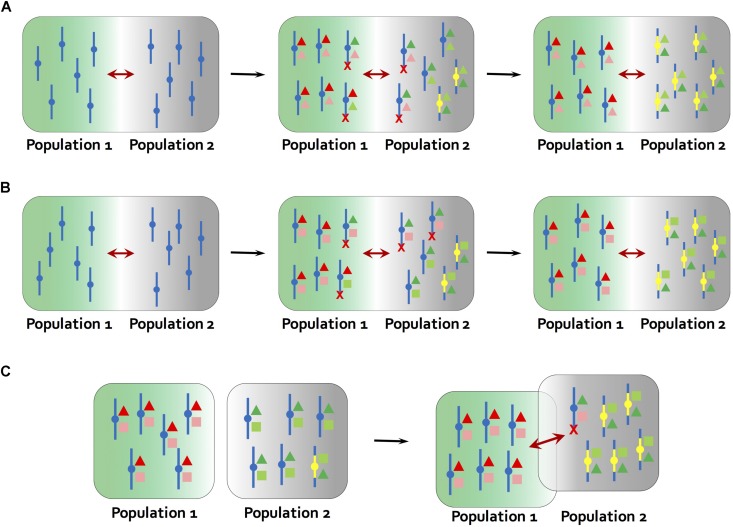
Models for the establishment of inversions. **(A)** Kirkpatrick and Barton (2006) model. At the starting point, population 1 and 2 occur in different environments, but are connected by gene flow (maroon arrows). Different alleles (red and green colors) at multiple genes underlying the same locally adapted trait (deep color and light color triangles) are favored in local environments (green and gray backgrounds). The ancestral chromosome carries mixtures of adapted and maladapted alleles in the face of gene flow, while a new inversion carries only the locally adapted alleles (yellow bars). The inversion is therefore favored and rises to high frequency in population 2. **(B)** Inversions become established through a process similar to **(A)** but by carrying a combination of alleles at two loci that are adapted to different aspects of the local environment (triangles and squares in different colors). For example, in a dune ecotype of the prairie sunflower (*Helianthus petiolaris*), larger seed size and tolerance to low nutrient soils were found to map to the same inversions (Huang et al., 2019; Todesco et al., 2019). **(C)** Mixed geographic model proposed by Feder et al. (2011). At the starting point, population 1 and 2 are allopatric. Multiple locally adapted alleles (triangles and squares in different colors) are fixed due to lack of gene flow, and an inversion carrying a full complement of these alleles is present at low frequency in population 2 through mutation-purifying selection balance or genetic drift. At secondary contact, the reduction in recombination caused by the inversion results in a selective advantage over collinear regions, leading to rise of inversion frequency. Red crosses indicate that chromosomes carrying maladaptive combinations of alleles are eliminated in each environment.

Newer models suggest that such epistatic interactions are not required if the inversions bring together two or more alleles that are adapted to the same local environment and there is ongoing migration between environments (Kirkpatrick and Barton, 2006; Figures 2A,B). In this situation, the newly derived inversion will have a selective advantage over the ancestral collinear arrangement that carries mixtures of adapted and maladapted alleles (Kirkpatrick and Barton, 2006; Burger and Akerman, 2011). However, a recent re-examination of the model showed that the selective advantage of an inversion will be small if the loci contained within the inversion are already tightly linked (Charlesworth and Barton, 2018). Thus, the conditions under which an inversion is favored in this model are less permissive that previously thought.

Empirical studies that associate multiple locally adapted traits or genes with inversions offer indirect support for this model. For example, Lowry and Willis (2010) showed that the chromosomal inversion differentiating annual and perennial ecotypes in *Mimulus guttatus* was associated with flowering time and morphological traits, as well as fitness in inland and coastal environments. Follow-up studies indicated that the inversion was associated with life history divergence and environmental variation, as well as adaptive trade-offs among growth, reproduction, and herbivore resistance (Oneal et al., 2014; Lowry et al., 2019). Similarly, in wild *Zea mays* an inversion on chromosome 1 showed a strong altitudinal cline in population frequency and statistical association with phenotypic traits such as culm diameter (Fang et al., 2012).

However, it is often unclear whether the inversions have captured pre-existing combinations of locally adapted alleles or whether such allelic combinations accumulated after inversion establishment. A number of studies in plants have successfully addressed this question, thereby offering more direct support for the Kirkpatrick and Barton (2006) model. Lee et al. (2017) made use of available collinear local adapted genotypes in *Boechera stricta* for genetic mapping and showed that pre-existing locally adaptive alleles may be captured by young inversions and contribute to local adaptation and incipient speciation. Likewise, Coughlan and Willis (2019) showed that key life history QTLs mapping to an inversion differentiating annual and perennial *Mimulus guttatus* mapped to the same region in a population involving annual *M. guttatus* and a collinear perennial species, *M. tilingii*, thereby showing that loci contributing to local adaptation predate the inversion in this system as well. Inversions on chromosome NC6 of *Noccaea caerulescens* are found to group pre-existing metal homeostasis genes, which may explain their fixation and role in speciation (Mandakova et al., 2015).

Inversion establishment in the Kirkpatrick and Barton (2006) model is also constrained by migration rates. Gene flow between different environments must be sufficiently high to generate a selective advantage for the new inversion. But high gene flow will lead to recombination between adapted and non-adapted alleles, reducing the likelihood that a new inversion would bring together a complete set of locally adapted alleles. A possible solution to this issue was suggested by Feder et al. (2011), who developed a mixed geographic model, in which adaptation to different environments occurs in allopatry, so that it is straightforward for a new inversion to capture a full cassette of adaptive alleles. Subsequent range expansion and secondary contact would give the new inversion a selective advantage over collinear regions (as in the Kirkpatrick and Barton model), leading to its establishment (Figure 2C). Given that range fluctuations are common in plants, and that this model permits inversion establishment from standing variation, we suspect that it might be a common mechanism.

**FIGURE 3:**
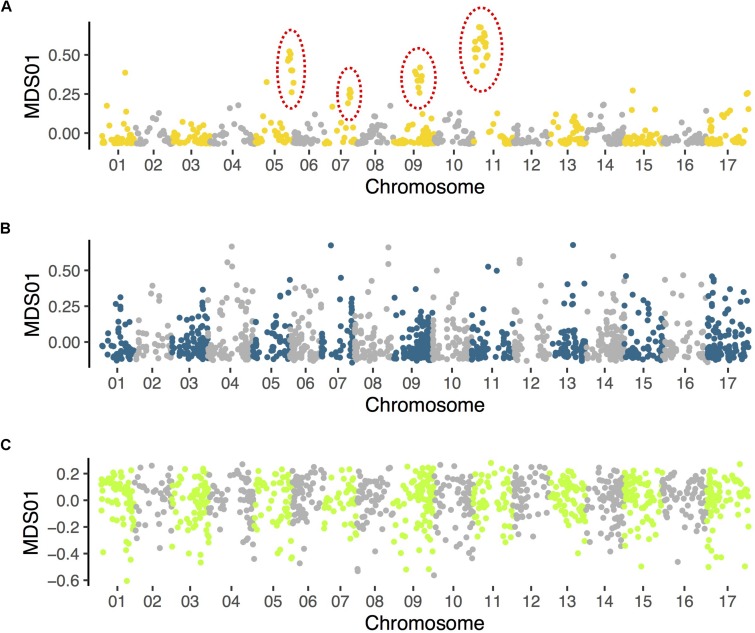
Results of local population structure analyses in **(A)** *Helianthus petiolaris* (data from Huang et al., 2019), **(B)** *H. bolanderi* (data from Owens et al., 2016), and **(C)** *H. niveus* (data from Zhang et al., 2019). Variant calling and multidimensional scaling (MDS) follow the same methods described in Huang et al. (2019). Only the first MDS coordinate is plotted. Clusters of MDS outliers, which indicate putative inversions (and have been confirmed with other methods), are identified in *H. petiolaris* (indicated with dotted circles) but not in the other two species.

Evidence that secondary contact promotes the spread of inversions has been found in birds (Hooper and Price, 2017), but to date there has been little relevant data in plants. However, new data from *Helianthus* sunflowers implies that secondary contact and hybridization may contribute importantly to the establishment of large inversions. We used a combination of population genomic, comparative mapping, and HiC sequencing to detect numerous polymorphic inversions within *Helianthus annuus*, *H. argophyllus* and *H. petiolaris* (Huang et al., 2019; Todesco et al., 2019), which are sympatric and known to hybridize with multiple other species. However, when we applied a similar population genomic approach to the analyses of two *Helianthus s*pecies that are largely (*H. bolanderi*; data from Owens et al., 2016) or completely (*H. niveus*; data from Zhang et al., 2019) allopatric, we failed to find clear signals of inversions (Figure 3). A mixed geographic model might explain why inversions are only found in *Helianthus* species that have extensive range overlap with others taxa.

Secondary contact can also shape current pattern of inversions within species. Phylogenomic analyses of inversions segregating within *Helianthus* species revealed that these inversion haplotypes typically are highly divergent, pre-dating the split between species (Todesco et al., 2019). While such a pattern could be due to balancing selection, the lack of trans-specific inversion polymorphisms (i.e., none of the inversions are polymorphic in more than one species), suggests that they might have been acquired from other, possibly extinct, species instead. This could have occurred via introgression or species fusion. Note that the latter would also account for the “extinction” of donor species. Evidence for the origin of inversions through introgression is known from animals (Feder et al., 2003b; Tuttle et al., 2016), but evidence in plants is slim. Clearly, phylogenomic analyses of inversion origins and ages in other plant groups should be a priority for future studies.

## Role in Speciation

Early models of chromosomal speciation were based on the assumption that inversions and other chromosomal rearrangements reduced gene flow between taxa through their effects on hybrid fitness. However, due to the paucity of evidence of reduced fitness in hybrids heterozygous for inversions, as well as the theoretical difficulties associated with fixing strongly underdominant mutations, another class of models was developed based on the effects of inversions on recombination rates within the inverted region (Trickett and Butlin, 1994; Noor et al., 2001; Rieseberg, 2001). These recombination suppression models offer a means for resolving the widely recognized antagonism between divergent natural selection and recombination, permitting adaptive divergence and speciation in the presence of gene flow (Ortiz-Barrientos et al., 2016).

**FIGURE 4:**
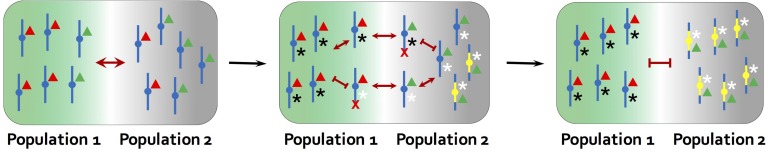
The model for the role of inversions in speciation proposed by Trickett and Butlin (1994). An inversion facilitates speciation by suppressing recombination between genes involved in local adaptation (red and green triangles) and those underlying assortative mating traits, as such flowering time (black and white asterisks). The ancestral chromosome carries mixtures of adapted and maladapted alleles due to recombination. Individuals that are locally adapted to the environment of population 2, but carries the white assortative mating allele, will tend to mate with individuals adapted to the other environment and produce maladaptive offspring in population 2. Individuals with a new inversion that captures only the locally adapted alleles and black assortative mating allele do not suffer the reproductive cost from recombination. The inversion is therefore favored and contributes to further divergence between populations. Red crosses indicate that chromosomes carrying maladaptive combinations of alleles are eliminated in each environment.

In this simplest model (Trickett and Butlin, 1994), an inversion reduces recombination between loci contributing to local adaptation and those causing assortative mating, permitting adaptive divergence and potentially speciation in the presence of gene flow (Figure 4). Such a genetic architecture appears to be common in plants. For example, the chromosomal inversion that contributes to local adaptation in inland and coastal environments in *Mimulus guttatus* is also associated with flowering time and other life history differences (Lowry and Willis, 2010; Oneal et al., 2014), thereby contributing to assortative mating between the annual and perennial ecotypes. In *M. lewisii* and *M. cardinalis*, inversions were also found to co-localize with a series of floral trait QTLs, such as corolla length and flower color, which are important in both prezygotic and postzygotic isolation in this species pair (Fishman et al., 2013). Similarly, in wild *Arabidopsis thaliana*, an inversion on chromosome 4 was reported to be strongly associated with fecundity under drought and an early flowering allele (Fransz et al., 2016). In *Boechera stricta*, Lee et al. (2017) found multiple linked phenology QTLs, including flowering differences, within an inversion that differentiates subspecies. In sunflower, inversions were associated with multiple ecological relevant traits, such as seed size and various soil and climate characteristics, as well as flowering time, revealing their role in ecotype formation and ecological speciation (Huang et al., 2019; Todesco et al., 2019).

While all of these examples demonstrate recombination suppression between locally adapted alleles and an assortative mating trait (most frequently flowering time), it is important to keep in mind that plant ecotypes and species often exhibit eco-geographic isolation. For example, in dune versus non-dune ecotypes of sunflower, the strongest reproductive barriers are immigrant inviability and extrinsic selection against hybrids (Ostevik et al., 2016). Strong selection against small seeds on the dunes, combined with a negative trade-off between seed size and seed number, underlies both barriers. Thus, isolation in this system is mainly due to a classic locally adapted trait (seed size), which maps to three inversions (Todesco et al., 2019), rather than assortative mating traits such as flowering time variation or conspecific pollen precedence. This situation is not unique to sunflowers and implies that the Trickett and Butlin (1994) model should be applicable to any trait that causes reproductive isolation, not just those contributing to assortative mating.

In addition to the Trickett and Butlin model, inversions have also been proposed as a means for maintaining hybrid incompatibilities in the face of ongoing gene flow (Noor et al., 2001), facilitating the accumulation of additional hybrid incompatibilities (Navarro and Barton, 2003), and extending the time window for reinforcement to evolve (Servedio, 2000, 2009). Also, by suppressing recombination, inversions can extend the effects of genes that contribute in some way to isolation over larger genomic regions (Rieseberg, 2001), thereby generating “genomic islands of divergence” (Oneal et al., 2014; Twyford and Friedman, 2015; Huang et al., 2019). Lastly, by combining the effects of multiple locally adapted alleles, the selective advantage of an inversion is expected to be greater than that of individual alleles, permitting divergence under higher migration rates (Rieseberg, 2001; Kirkpatrick and Barton, 2006). However, of these potential roles, only the association of inversions with genomic islands of divergence has been documented in plants.

## Sex Chromosome and Sequential Inversions

Although most plants are hermaphrodite (co-sexual), some plant species have evolved separate male and female sex morphs (i.e., dioecy). The transition from a co-sexual breeding system to dioecy typically involves the formation of sex chromosomes, in which recombination suppression evolves between male and female sterility loci (Charlesworth, 2012). Inversions offer a straightforward means for suppressing recombination between newly formed X and Y (or Z and W) chromosomes and have been reported in numerous animal systems, as well as in a handful of plant species. For example, two large inversions were found to define the non-recombining region between Y and X chromosomes in papaya, suggestive of a role in sex chromosome formation (Wang et al., 2012).

The evolution of sexually antagonistic genes, which are favored in one sex but not in the other, provides additional selection pressure to reduce recombination between sex chromosome pairs; otherwise antagonistic alleles will be transmitted to the opposite sex. As a consequence, over time recombination suppression typically expands to cover most of the sex chromosome pair. Interestingly, in many animals and some plant species, such expansions appear to be episodic, producing “evolutionary strata” across sex chromosomes, i.e. spatial clusters of X-Y or Z-W orthologs with similar divergence estimates (Wright et al., 2016). Such strata are often closely associated with inversions, leading to suggestions that the stepwise establishment of inversions might be responsible for this pattern of divergence. Currently, the best evidence for this hypothesis in plants comes from papaya, in which two distinct evolutionary strata were discovered that correspond perfectly with the boundaries of the two inversions (Wang et al., 2012). In *Silene*, both strata (Nicolas et al., 2004; Bergero et al., 2007) and inversions (Zluvova et al., 2005; Hobza et al., 2007) have been reported, but they are not explicitly linked. Other authors have noted that recombination suppression associated with sex chromosome divergence sometimes occurs through other mechanisms such as transposable element insertion (e.g., Xu et al., 2019). Therefore, it is possible that suppressed recombination comes first, followed by the accumulation of inversions. An example in which recombination suppression precedes chromosomal rearrangements has been reported in fungi (Sun et al., 2017), but as far as we are aware, evidence for such a scenario is lacking in plants.

The stepwise accumulation of inversions need not be restricted to sex chromosomes. An inversion on an autosome could initially become established by capturing multiple locally adapted alleles, as proposed in the Kirkpatrick and Barton (2006) model. Subsequent inversions that added new locally adapted alleles into the non-recombining block would be favored by selection. This stepwise extension of recombination suppression presumably would create evolutionary strata similar to that seen on sex chromosomes. The apparent clustering of inversions seen in comparisons between cucumber and melon (Garcia-Mas et al., 2012), as well as between domesticated rice and *Oryza brachyantha* (Chen et al., 2013), are consistent with this hypothesis. Future dissection of the structure and divergence patterns of complex inversions should be a priority.

## Concluding Remarks

Since the discovery of inversions in *Drosophila* close to a century ago, numerous verbal and quantitative models have explored their potential role in evolution and possible mechanisms for their establishment. Comparative genetic mapping and genomic studies have revealed that chromosomal inversions are far more prevalent than previously imagined. However, these studies often focus on large inversions only and/or fail to report on inversion sizes or the extent of sequence divergence between inversion haplotypes. These information gaps should be addressed in future comparisons of reference assemblies.

Likewise, an ever-increasing number of studies in plants suggest that inversions play a key role in adaptive divergence and speciation in the presence of gene flow. However, the genes and mutations underlying key traits associated with the inversions are difficult to identify because of strong linkage disequilibrium within the inverted region. Analyses of collinear genomes that are expected to differ for many of the same genes will aid in this process, as shown by two examples here. Population genetic and molecular tools would also help pinpoint the genetic changes within inversions that are responsible for adaptive differences or speciation. Of particular interest are regions near inversion breakpoints, since the inversions have the potential not only to disrupt open-reading frames or associations with regulatory elements, but also to change the local chromosome landscape of genes (e.g. potentially moving genes closer or further away from heterochromatic regions). Lastly, little is known in plants about potential downsides of inversions such as increased transposable element activity and the accumulation of deleterious mutations, both of which are a predicted consequence of suppressed recombination. This information gap that could also be addressed with population genomic analyses.

Despite the rapid accumulation of examples of the importance of inversions in a variety of ecological and evolutionary processes, information on their origin is scarce. Inversions can become established in several ways, but models based on the advantages of reducing recombination between locally adapted alleles when there is migration between different environments seem most plausible. This process is likely aided in some instances by a period of allopatry between hybridizing populations so that the full set of locally adapted alleles can be captured by the new inversion. Phylogenomic analyses of closely related species are needed to determine the origins and extent of divergence between inversion haplotypes, since evidence suggests that hybridization and introgression may contribute to inversion establishment and subsequent evolutionary dynamics. Lastly, we urge students of chromosomal evolution to assess whether inversions are clustered in the genome and if evolutionary strata can be discovered on autosomes, similar to what has been reported for sex chromosomes.

## Author Contributions

LR conceived the idea. KH performed literature review, analyzed the data, and made the graphics. KH and LR wrote the manuscript.

## Conflict of Interest

The authors declare that the research was conducted in the absence of any commercial or financial relationships that could be construed as a potential conflict of interest.
